# The virulent *Wolbachia* strain wMelPop increases the frequency of apoptosis in the female germline cells of *Drosophila melanogaster*

**DOI:** 10.1186/1471-2180-12-S1-S15

**Published:** 2012-01-18

**Authors:** Mariya V Zhukova, Elena Kiseleva

**Affiliations:** 1 Institute of Cytology and Genetics, Siberian Branch of the Russian Academy of Sciences, Novosibirsk, 630090 Russia

## Abstract

**Background:**

*Wolbachia* are bacterial endosymbionts of many arthropod species in which they manipulate reproductive functions. The distribution of these bacteria in the *Drosophila* ovarian cells at different stages of oogenesis has been amply described. The pathways along which *Wolbachia* influences *Drosophila* oogenesis have been, so far, little studied. It is known that *Wolbachia* are abundant in the somatic stem cell niche of the *Drosophila* germarium. A checkpoint, where programmed cell death, or apoptosis, can occur, is located in region 2a/2b of the germarium, which comprises niche cells. Here we address the question whether or not the presence of *Wolbachia* in germarium cells can affect the frequency of cyst apoptosis in the checkpoint.

**Results:**

Our current fluorescent microscopic observations showed that the wMel and wMelPop strains had different effects on female germline cells of *D. melanogaster.* The *Wolbachia* strain wMel did not affect the frequency of apoptosis in cells of the germarium. The presence of the *Wolbachia* strain wMelPop in the *D. melanogaster^w1118^* ovaries increased the number of germaria where cells underwent apoptosis in the checkpoint. Based on the appearance in the electron microscope, there was no difference in morphological features of apoptotic cystocytes between *Wolbachia*-infected and uninfected flies. Bacteria with normal ultrastructure and large numbers of degenerating bacteria were found in the dying cyst cells.

**Conclusions:**

Our current study demonstrated that the *Wolbachia* strain wMelPop affects the egg chamber formation in the *D. melanogaster* ovaries. This led to an increase in the number of germaria containing apoptotic cells. It is suggested that *Wolbachia* can adversely interfere either with the cystocyte differentiation into the oocyte or with the division of somatic stem cells giving rise to follicle cells and, as a consequence, to improper ratio of germline cells to follicle cells and, ultimately, to apoptosis of cysts. There was no similar adverse effect in *D. melanogaster* Canton S infected with the *Wolbachia* strain wMel. This was taken to mean that the observed increase in frequency of apoptosis was not the general effect of *Wolbachia* on germline cells of *D. melanogaster*, it was rather induced by the virulent *Wolbachia* strain wMelPop.

## Background

Apoptosis, a form of programmed cell death, is a process needed for normal development and maintenance of tissue homeostasis in multicellular organisms [1,2]. Cells undergoing apoptosis show characteristic changes, such as chromatin and cytoplasm condensation, chromosomal DNA fragmentation, breaking up of nuclei and then of cells into fragments called apoptotic bodies [3,4]. Individual cells apoptose, while the neighboring cells remain undamaged [3,4]. Apoptosis is a complex process whereby a proteolytic cascade of caspases is activated in cells [5].

The occurrence of apoptosis is a feature of female germline development common to vertebrate and invertebrate species [6,7]. In the *Drosophila melanogaster* ovaries, there are two checkpoints where programmed cell death occurs. One is in the germarium (region 2a/2b), where apoptosis probably regulates the proper ratio of germline cells to follicle cells [8]. The other checkpoint is located in the vitellarium (stages 7-8 of oogenesis) [9]. The number of egg chambers undergoing apoptosis increased in *D. melanogaster* fed a diet lacking protein [8], under the effect of 900-MHz and 1800-MHz radiation [10], and after exposure to chemical agents [11]. The normal development of mature egg is consistently associated with apoptosis of 15 nurse cells in the egg chamber [12]. It is noteworthy that apoptosis and autophagy coexist at all the above mentioned stages of oogenesis in *D. melanogaster *[13,14].

It has been also hypothesized that the apoptotic process had a symbiotic origin [15]. In terms of the endosymbiotic theory, mitochondria, which play a major role at the early stages of apoptosis, evolved from the free-living prokaryotes [5]. One of the symbionts may be involved in the regulation of apoptosis in partner cells. To illustrate, extracellular parasites, particularly such worms as filarial nematodes, schistosomes and the cestode *Taenia crassiceps*, are able to induce apoptosis in host immune cells [16]. Bacterial pathogens (*Chlamydia*, *Neisseria*, *Legionella pneumophila*) can either block or induce apoptosis in host cells, depending on the stage of infection [17,18]. At the early stage of infection, bacteria replicate in the host cell, using different mechanisms to prevent apoptosis. At the late stages of infection, the bacteria induce apoptosis in the host cell, thereby facilitating egress and ensuring infection of neighboring cells.

*Wolbachia* associated with various hosts in which it manipulates viability and reproduction causing parthenogenesis, feminization, male killing and cytoplasmic incompatibility, provides a unique model for studying mechanisms of symbiont interactions [19,20]. The *Wolbachia* strain wMel is widely spread in natural populations of *D. melanogaster *[21,22]; in contrast, wMelPop has been detected in a laboratory stock of *D. melanogaster *[23]. It is possibly not encountered in nature. In *D. melanogaster*, the wMelPop strain reduces lifespan, proliferating widely in the brain, muscle and retina cells [23]. In certain insect species, the presence of *Wolbachia* is required for oogenesis [24]. Removal of the *Wolbachia* strain wAtab3 from parasitic wasps *Asobara tabida* resulted in copious apoptosis of the egg chambers in the ovarioles and led to sterility [25]. The mechanisms whereby the endosymbiont *Wolbachia* impacts apoptosis in host cells have been poorly studied. Preferential infection and high accumulation of *Wolbachia* in region 2a of the germarium [26] where the checkpoint is located in *Drosophila* was thought-provoking. We raised the question: Can bacteria *Wolbachia* in region 2a of the germarium affect the frequency of apoptosis there? Using fluorescence and transmission electron microscopy we compared germaria from ovaries of two *D. melanogaster* stocks infected with either the wMel or wMelPop strains with germaria from two uninfected counterparts. It was established that the presence of wMel did not alter apoptosis frequency in germaria from *D. melanogaster* Canton S. In contrast, the number of germaria containing apoptotic cells in the checkpoint was considerably increased in the wMelPop-infected flies as compared with their uninfected counterparts. Thus, evidence was obtained indicating that the virulent *Wolbachia* strain wMelPop has an effect on the fate of germline cells during *D. melanogaster* oogenesis.

## Results

### Frequency of apoptosis in germaria from ovaries of the uninfected and *Wolbachia*-infected *D. melanogaster*

**Figure 1:**
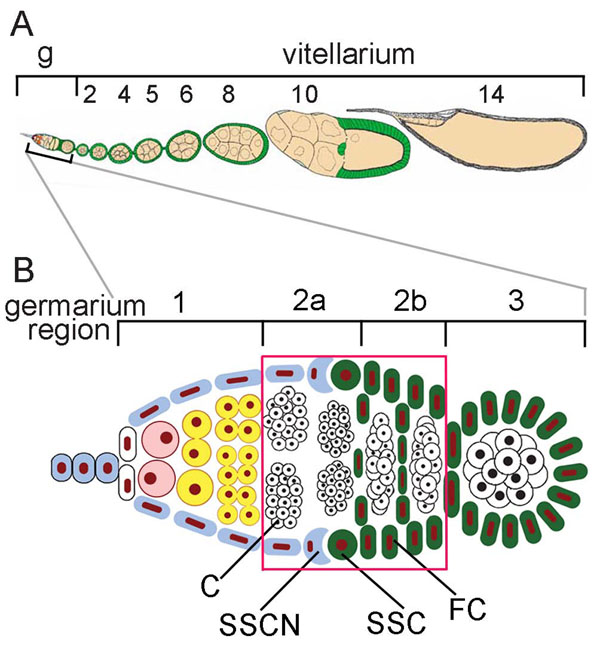
**A schematic representation of an ovariole of *D. melanogaster*.** A, an ovariole of *D. melanogaster* consisting of the germarium (g) and the vitellarium. B, a detailed scheme of the germarium structure composed of regions 1, 2a, 2b, 3. The checkpoint is framed (red). C, a 16-cell cyst; SSCN, a somatic stem cell niche; SSC, a somatic stem cell; FC, a follicle cell.

**Figure 2:**
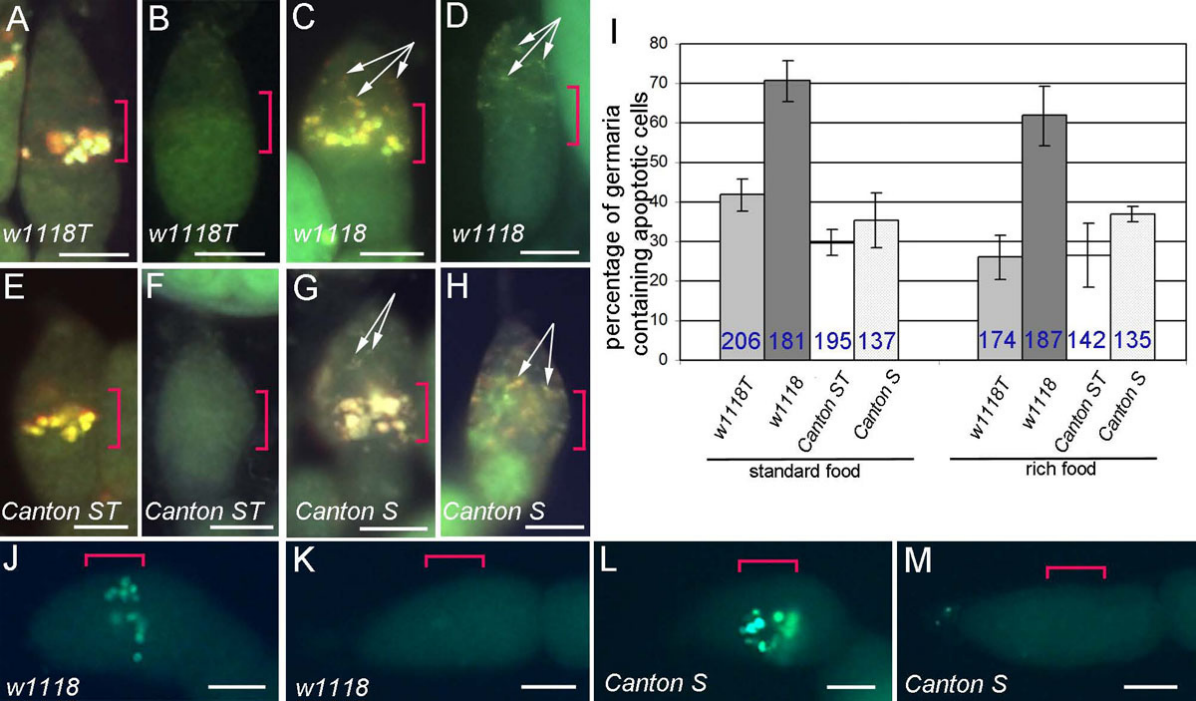
**Visualisation of acridine orange (AO)- and TUNEL-stained germarium cells of *D. melanogaster*.** A, C, E, G, germaria containing apoptotic cells in region 2a/2b from 5 day-old uninfected (A, E) and *Wolbachia*-infected (C, G) females (AO staining). B, D, F, H, germaria not containing apoptotic cells from the same fly stocks (AO staining). Arrows indicate small punctate AO-staining in regions 1 and 2a/2b (C, D, G, H). I, relative proportion of germaria containing apoptotic cells from ovaries of the uninfected (w1118T, Canton ST) and *Wolbachia*-infected (w1118, Canton S) flies. The total number of examined germaria is indicated by blue number; bars show the average percentage per experiment ± s. e. m. J, L, germaria containing apoptotic cells in region 2a/2b in the wMelPop- and wMel-infected fly stocks, respectively (TUNEL). K, M, germaria not containing apoptotic cells from the same fly stocks. Region 2a/2b of the germarium is indicated by red brackets. Scale bars: 20 μm.

Two parts are distinguished in the *Drosophila* ovariole: the germarium made up of four regions (1, 2a, 2b, 3) and the vitellarium (Figure 1A, B) [27,28]. The region 2a/2b, where apoptosis can occur, contains 16-cell cysts, somatic stem cells (SSCs), which contact with the somatic stem cell niche (SSCN) and follicle cells (Figure 1B). Cell death in this region of the germarium was detected by two methods, acridine orange (AO)-staining and TUNEL assay. Fluorescence microscopy of AO-stained ovarioles demonstrated that apoptotic cells were located as large yellow or orange spots in region 2a/2b of the germarium from *D. melanogaster* (Figure 2A, C, E, G). Germaria containing no apoptotic cells fluoresced homogeneous green (Figure 2B, D, F, H). It should be noted that wMel- and wMelPop-infected flies, besides bright spots in region 2a/2b (Figure 2C, G), showed weak punctuate fluorescence both in regions 2a/2b and 1 of the germarium (Figure 2C, D, G, H). Such fluorescent puncta were not observed following TUNEL, thereby indicated that they were not caused by apoptosis.

The percentage of germaria containing apoptotic cells was 41.8±4.1% in the uninfected *D. melanogaster^w1118T^* maintained on standard food, whereas it increased to 70.6±5.3% in the wMelPop-infected flies (Figure 2I). Analysis performed with the wMel-infected *D. melanogaster* Canton S revealed no significant differences from their uninfected counterparts (Figure 2I, Table 1).The next step was to exclude the possible effect of insufficient nutrition on the current results. To do so, we conducted experiments in which flies were raised on rich food source taking into account that it decreases the number of germaria containing apoptotic cells [8,29]. We found that rich food causes a decrease in the relative proportion of apoptotic germaria in both *w^1118T^* and *w^1118^* flies; however, the difference between these two groups was significant (Figure 2I, Table 1). The percentage of germaria containing apoptotic cells did not change under the effect of rearing *D. melanogaster* Canton S on different food. Based on analysis of apoptotic cell death by TUNEL, three groups of germaria were distinguished: TUNEL-negative, TUNEL-positive with 1-2 distinct puncta in region 2a/2b and TUNEL-positive with a cluster of bright spots (Additional file 1). There was no evidence for variation in the frequency of apoptosis between wMel-infected (Canton S) and uninfected (Canton ST) flies (Table 2; χ^2^=1.3, df=1, P=0.25); however, there was evidence for a difference in the frequency of apoptosis between the *w^1118T^* and *w^1118^* flies (Table 2; χ^2^=25.3, df=1, P<0.0001). The total percentage of germaria containing apoptotic cells in *D. melanogaster* agreed well with the one obtained with AO-staining. Thus, TUNEL confirmed the results of AO-staining.

**Table 1:** Details of statistical analysis (two-way ANOVA)

Source of variation	Canton S/Canton ST		w^1118^/ w^1118T^	
	
	% of total variation	P value	% of total variation	P value
Interaction	1,51	0,7065	0.74	0,4998
Type of food	0,15	0,9045	9,23	0,0312
Infection status	19,30	0,1998	63,68	P<0.0001

Data of AO-staining of germaria from 4 fly stocks maintained at different food.

**Table 2:** Quantification of TUNEL staining in region 2a/2b of the germaria

Fly stock	TUNEL-negative	TUNEL-positive		Total % of TUNEL-positive	Total number
		
		Distinct puncta	Cluster of spots		
Canton ST	61.6% (133)	8.8% (19)	29.6% (64)	38.4%	216
Canton S	56.3% (134)	10.1% (24)	33.6% (80)	43.7%	238
*w^1118^*^T^	59.1% (111)	13.8% (26)	27.1% (51)	40.9%	188
*w^1118^*	34.6% (82)	14.3% (34)	51.1% (121)	65.4%	237

### Ultrastructure of germaria from ovaries of the uninfected and the *Wolbachia*-infected *D. melanogaster*

**Figure 3:**
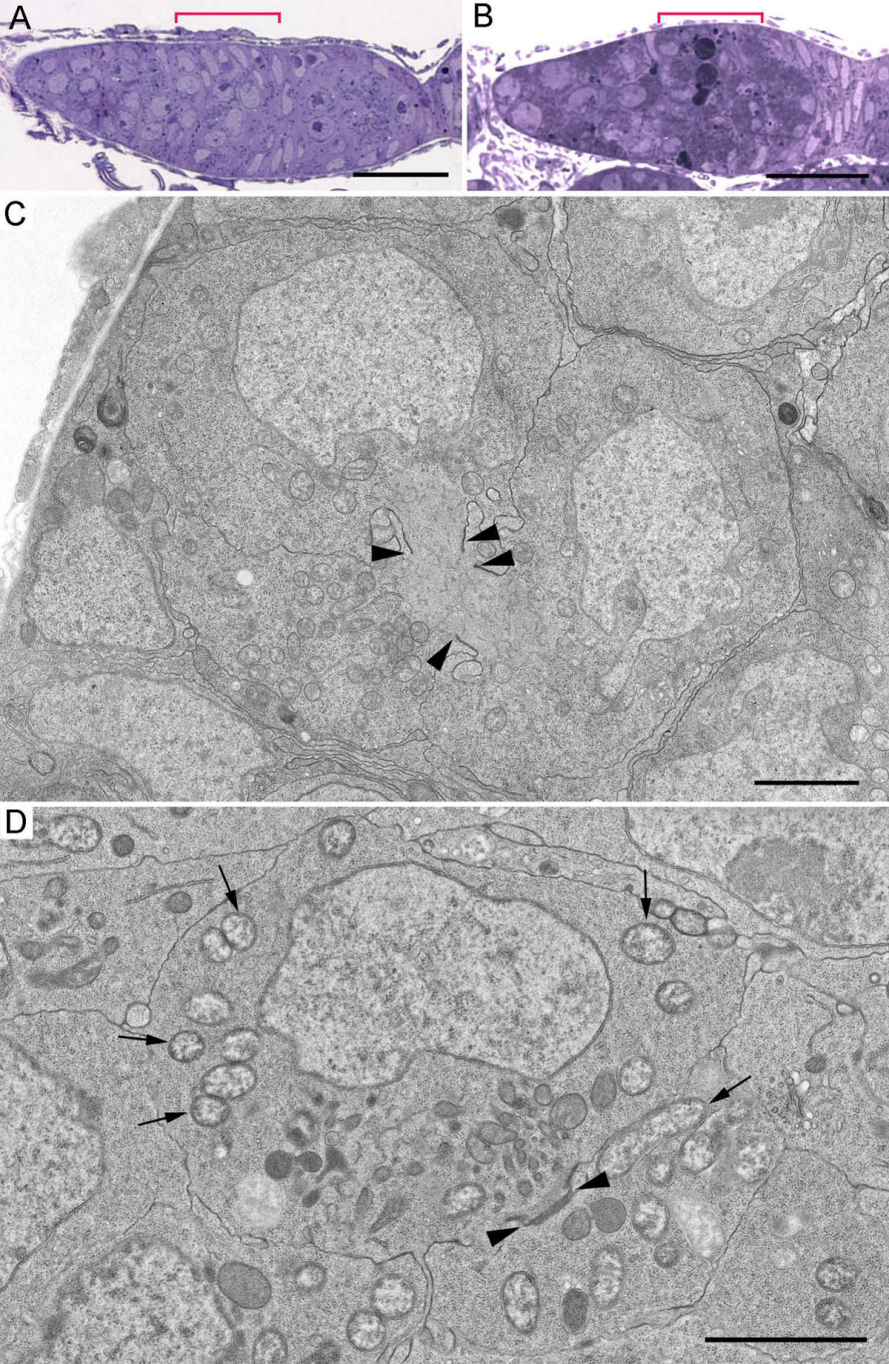
**Visualisation of germarium cells in semi-thin and ultra-thin sections.** A, B, longitudinal semi-thin sections of germaria stained with methylene blue. C, D, ultrastructure of cyst cells from the uninfected and the wMelPop-infected flies. Arrows point to bacteria; arrowheads denote ring canals between neighboring cells. Scale bars correspond to 10 μm (A, B) and 2 μm (C, D), respectively.

**Figure 4:**
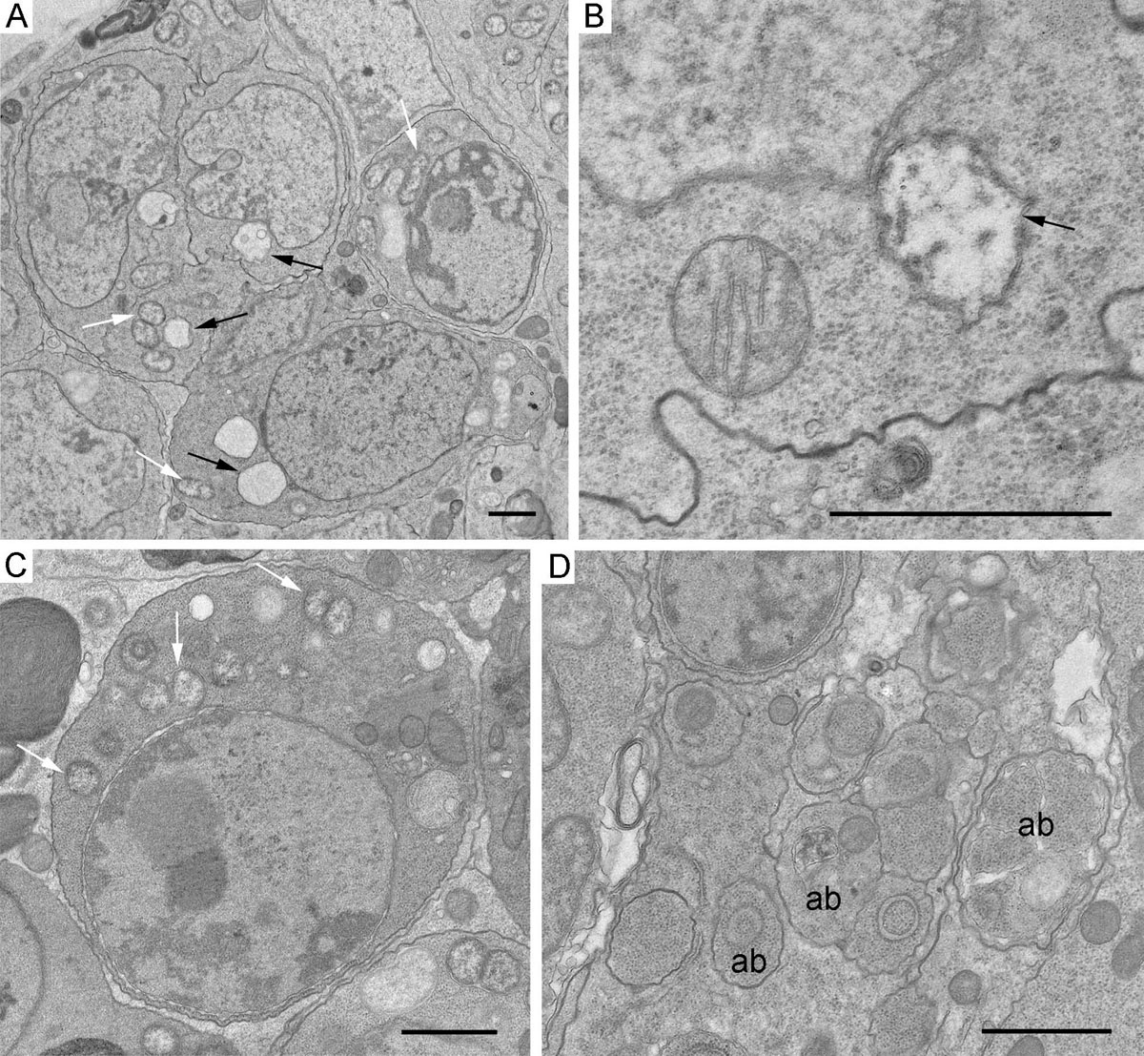
**Morphology of apoptotic cystocytes in region 2a/2b of the germarium from the wMelPop-infected *D. melanogaster^w1118^*.** A, swollen mitochondria (black arrows) in the cytoplasm of cyst cells. White arrows indicate bacteria. B, a fragment of a cyst cell with two mitochondria: one is normal, the other is swollen with the matrix of low electron density and the disintegrated cristae. C, a cyst cell, the cytoplasm appears dense, the nucleus is pyknotic. D, apoptotic bodies (ab) containing intracellular organelles. Scale bars: 1 μm.

For an ultrastructural analysis of germarium cells, we first chose under the light microscope those longitudinal sections that enabled us to define region 2a/2b of the germarium (Figure 3A, B, red brackets). Cyst cells in region 2a/2b were interconnected by ring canals and consisted of nuclei that exhibited numerous invaginations, protrusions, and cytoplasm rich in organelles (Figure 3C, D, Additional file 2). Our ultrastructural data for germarium cells of the uninfected and the *Wolbachia*-infected flies allowed us to identify cysts in region 2a/2b showing characteristic features of apoptotic death (Figure 4 and Additional file 3). The cytoplasm was more electron-dense in such cystocytes, some mitochondria became markedly swollen (Figs. 4A and Additional file 3A). The matrix of mitochondria was light and just a few small cristae were discerned at the periphery (Figs. 4B and Additional file 3B). We observed also cells with electron-dense cytoplasm, which had lost contact with their neighboring cells (Additional file 3C). In such cells, chromatin appeared condensed in apoptotic nuclei and the lumen of the nuclear envelope was dilated (Figs. 4C and Additional file 3C). At the last stage of apoptosis, cells disaggregated into large and small fragments, or apoptotic bodies, with characteristic electron-dense cytoplasm containing ribosomes, endoplasmic reticulum membranes, and frequently intact mitochondria (Figs. 4D and Additional file 3D).

**Figure 5:**
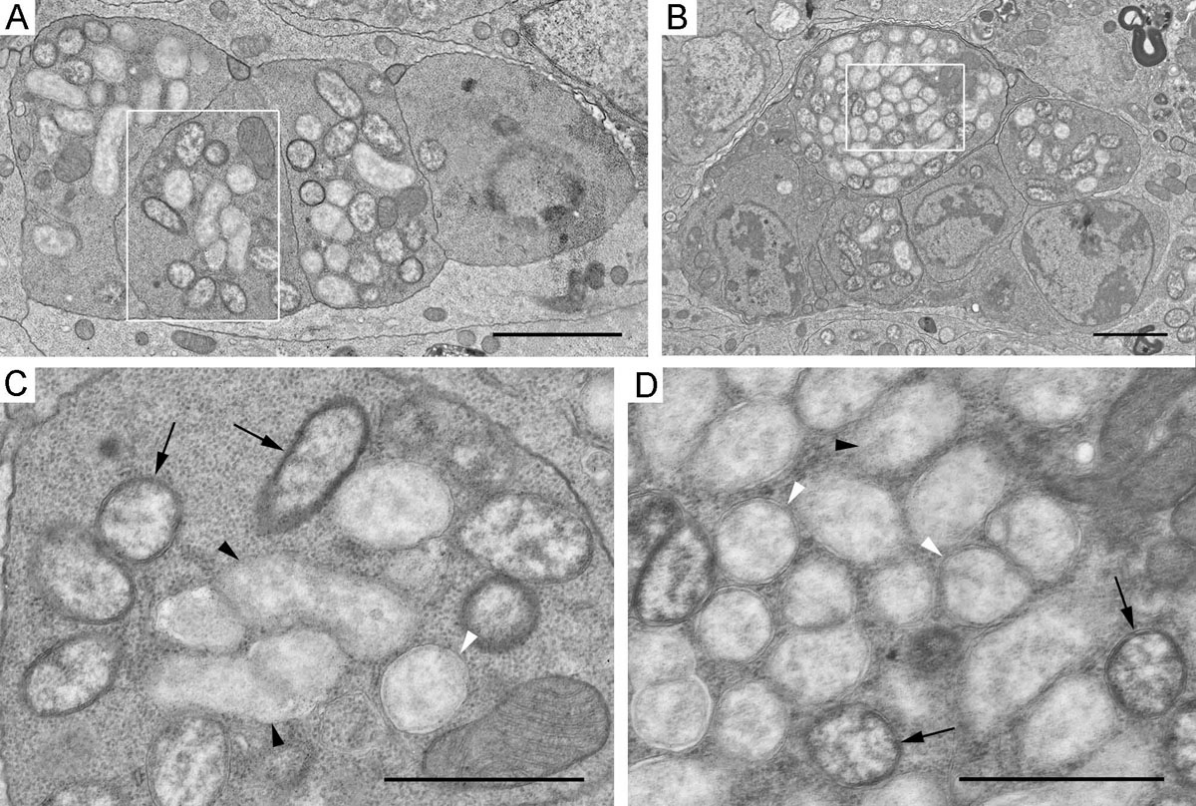
**Ultrastructure of the *Wolbachia* strain wMelPop in apoptotic cystocytes in region 2a/2b of the germarium.** A, B, *Wolbachia* accumulations in apoptotic cyst cells, low magnification view. C,D, bacteria framed in panels A, B depicted at higher magnification. Bacteria showing normal morphology (arrows), bacteria with matrix of low electron density (white arrowheads), bacteria with matrix of low electron density and disrupted cell wall (black arrowheads) in the cytoplasm of dying cysts. Scale bars: 2 μm.

Analysis of germarium cystocytes of wMel- and wMelPop-infected flies showed that individual bacteria were distributed throughout all the cytoplasm, occasionally occurring as small groups (Figs 3D and Additional file 2). Large accumulations of adjacent bacteria were observed in apoptotic cyst cells (Figure 5A, B, Additional file 4). In these masses, the bacteria varied in morphological appearance (5C, D and Additional file 4B). Some endosymbionts showed normal ultrastructural features: a three-layered envelope, a matrix with many ribosomes and dispersed chromatin. In contrast, most bacteria were surrounded by a three-layered envelope, the matrix was of low electron density with a few ribosomes. Disrupting bacteria were also encountered. These were not enclosed by an envelope, their matrix was loose, light, devoid of ribosomes. The follicle cells surrounding the cysts in region 2b of the germarium showed a normal morphology and low levels of *Wolbachia* with normal structure (Additional file 5).

**Figure 6:**
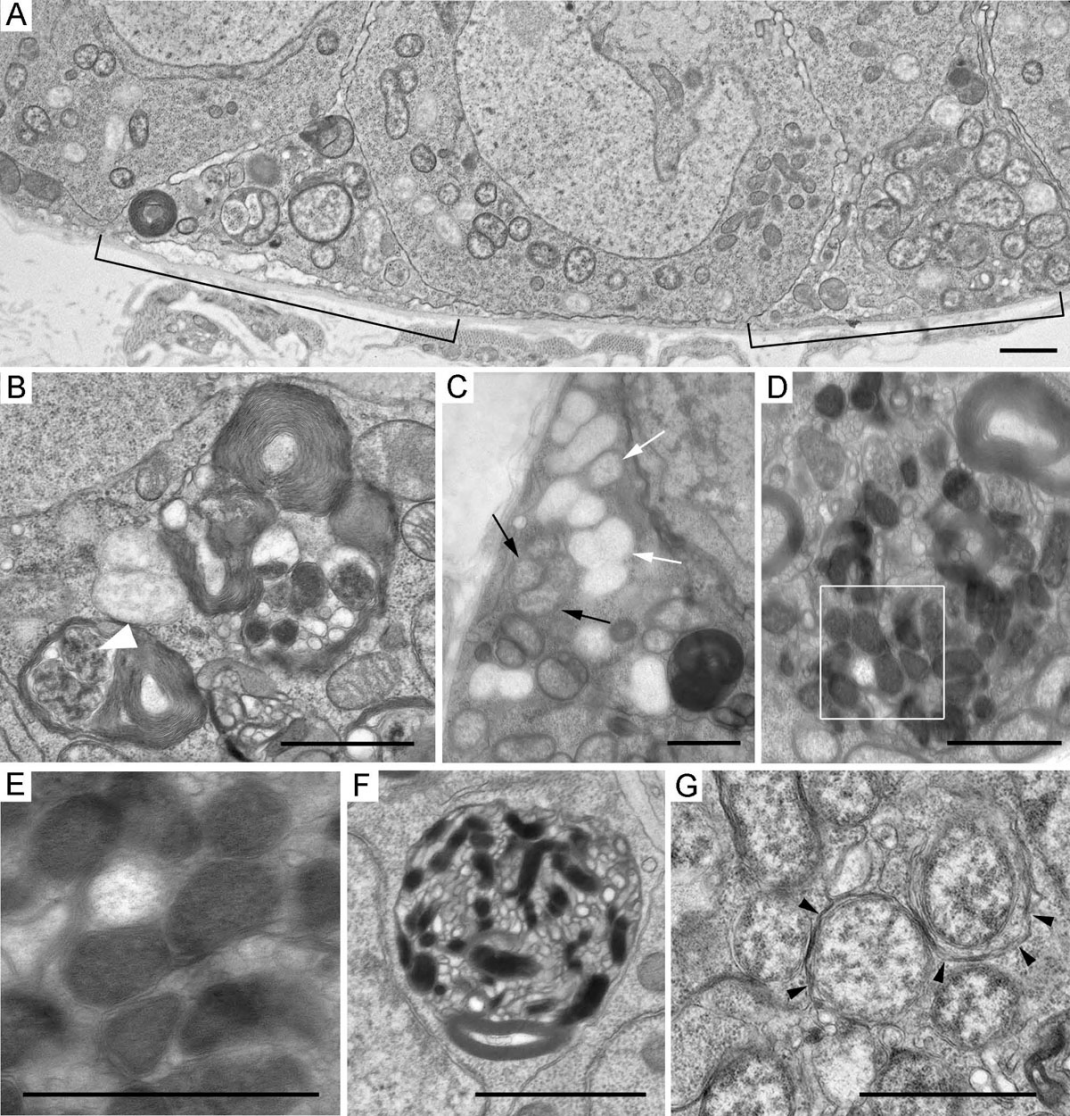
**Ultrastructure of the germarium cells at the periphery of region 1 in wMelPop-infected *D. melanogaster^w1118^*.** A, a fragment of region 1 of the germarium, low magnification view. Normal cells and two fragments of cells (brackets), whose cytoplasm is filled with autophagosomes, bacteria and multilayered membranes. B, multilayered membranes and fragments of a disintegrated nucleus (white arrowhead). C, a fragment of a cell with electron-dense cytoplasm containing *Wolbachia* of two types: one normal (black arrows), the other with matrix of low density (white arrows). D, electron-dense bacteria-like structures engulfed by autophagosome. E, higher magnification of the bacteria-like structure framed in panel D. F, an autophagosome containing electron-dense structures and vesicles . G, autophagosomes enclosed individual bacteria. Arrowheads indicate autophagosome membranes. Scale bars: 1 μm.

At the periphery of the germarium, fragments of degrading cells were frequently seen in region 1, precisely where AO-staining of the germaria from the *Wolbachia*-infected flies was punctate (Figure 2C, D, G, H). These fragments were filled with multilayered membranes, nuclear remnants, mitochondria, and bacteria with normal and abnormal morphology (Figure 6A-C, Additional file 6). The cell organelles and bacteria were often engulfed by autophagosomes. Besides bacteria with light matrix, like those in apoptotic cysts (Figure 6C, D), the autophagosomes occasionally enclosed electron-dense bacteria-like structures 0.2-0.3 μm in diameter (Figure 6D, E) or similar smaller ones (Figure 6F). At the periphery of the germaria, autophagosomes containing individual bacteria with normal morphology were observed (Figure 6G).

## Discussion

This is, to our knowledge, the first study that demonstrated by using AO- and TUNEL staining an increase in the frequency of apoptosis in the germarium checkpoint in wMelPop-infected *D. melanogaster^w1118^*. This increase is possibly caused by the specific effect of the *Wolbachia* strain wMelPop, since it was not observed in wMel-infected *D. melanogaster* Canton S. Our current electron microscopic observations allowed us to identify changes in *Wolbachia* morphology in apoptotic germline cells.

### Morphological evidence of apoptosis in germarium cells

The ultrastructural features of apoptosis in the cyst cells of higher eukaryotes have gained wide recognition. They include cytoplasmic and nuclear condensation (pyknosis); nuclear fragmentation (karyorrhexis); normal morphological appearance of cytoplasmic organelles; an intact plasma membrane [3,4]. The ultrastructural changes we identified here in *D. melanogaster* cyst cells are consistent with the above hallmarks. Furthermore, we revealed mitochondria of two types: intact morphology in one type and markedly swollen with a few cristae in the other. A similar heterogeneity of mitochondrial ultrastructure has been observed during apoptosis in granulose cells of Japanese quail (*Coturnix coturnix japonica*) [30], lymphocytes from leukemia patients [31], and megakaryocytes from patients with idiopathic thrombocytopenic purpura [32]. It has been suggested that the swollen mitochondria release cytochrome c, which activates a cascade of proteolytic reactions, while the normal ones retain their capacity for ATP synthesis, a process apoptosis requires [30,31,33]. According to our qualitative analysis using EM, morphological evidence of apoptosis was revealed in germline cells from uninfected flies and those infected with wMel and wMelPop. Thus, there are reasons for inferring that the endosymbiont *Wolbachia* in *D. melanogaster* cystocytes has no effect on sequential passage of intracellular organelles through apoptosis. To reveal the possible differences between the effect of the wMel and wMelPop strains on apoptosis in the germaria, additional morphometric analysis of the number of apoptotic structures and of *Wolbachia* density in the cystocytes is required.

### Structural features of *Wolbachia* in apoptotic cysts

*Wolbachia* with matrix of moderate and low electron density in apoptotic cells in region 2a/2b of the germarium have been previously encountered in other types of *D. melanogaster* ovaries [34] and they presumably reflect different functional states of bacteria. *Wolbachia* with disrupted envelopes and light matrix are possibly dying bacteria in apoptotic cells. Such appearance has not been observed in *Wolbachia* injured or killed by heat stress [35] and tetracycline [36]. The electron-dense bacteria-like structures at the periphery of region 1 of the germarium may be evidence of changes in dying *Wolbachia*. Large masses of structures of this kind resembling the bacteria endospores have been found in the brain cells of the wMelPop-infected *D. melanogaster^w1118^*[23]. In our view, the electron-dense structures, which we revealed at the periphery of region 1 of the germarium, are presumably autophagosome encapsulated dying *Wolbachia*. A supporting line of evidence came from Wright and Barr [37], who on the basis of their observations on degenerating germaria cysts from mosquitoes *Aedes scutellaris* suggested that these structures represented degenerating *Wolbachia*.

Cell fragments containing dying bacteria and autophagosomes and appearing as numerous smaller puncta in regions 2a/2b and 1 of the germarium may represent autophagy, not apoptosis. This appears plausible when recalling that AO stains not only apoptotic cells, also lysosomes [38]. TUNEL did not reveal such puncta in these regions.

### The possible role of the *Wolbachia* strain wMelPop in programmed cell death in region 2a/2b of the germarium

Our current estimates of apoptosis in region 2a/2b of the germarium from the ovaries of the uninfected *D. melanogaster^w1118T^* raised on standard food are consistent with those reported elsewhere [14]. It is of interest that apoptosis level in the germaria decreased in *D. melanogaster^w1118T^*, but not in *D. melanogaster* Canton ST after transfer to rich food. This may be indicative of differences in sensitivity to changes in food composition between different fly stocks. AO- and TUNEL staining demonstrated that the virulent *Wolbachia* strain wMelPop increased the percentage of germaria containing apoptotic cells in *D. melanogaster^w1118^* ovaries, while wMel strain was without such an effect. The effect of wMelPop on cystocytes in ovaries was observed in flies maintained on standard and rich food. Evidence was provided that the effect of *Wolbachia* on *D. melanogaster* is not general, being rather specific to the pathogenic strain wMelPop.

**Figure 7:**
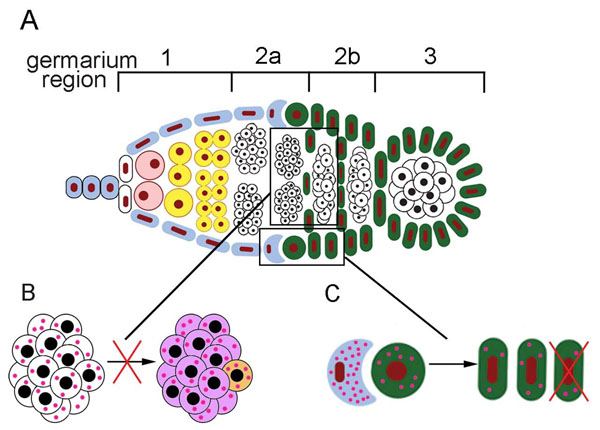
**Pathways along which *Wolbachia* may affect egg chamber formation in region 2a/2b of the germarium.** A, localization of regions in the germarium (framed) where the bacteria may interfere with normal function of cells. B, the bacteria disturb the differentiation of cystocytes (white) into the oocyte (light orange) and the nurse cells (light violet). C, the bacteria skew the proper ratio of germline cells to follicle cells. Crescent shape, SSCN; green circle, SSC; green ovals, follicle cells. Red points represent the bacteria.

What pathways may be envisaged for the *Wolbachia* strain wMelPop caused increase in the number of germaria whose cysts undergo apoptosis? On the one hand, bacteria may have a direct effect on germline cells (Figure 7A, B). In fact, one of 16 cyst cells becomes the oocyte, the other 15 differentiate into nurse cells in region 2a of the germarium. This is associated with transport of 15 centrioles into the pre-oocyte, where the microtubule-organizing center forms [39,40]. *Wolbachia* distribution is dependent upon microtubules during oogenesis and bacteria show mislocalization in the egg chambers treated with colchicine which causes depolymerization of microtubules [41]. Evidence has been obtained indicating that *Wolbachia* are evenly distributed throughout the oocyte and nurse cells during stages 1-2 of oogenesis, becoming concentrated at the oocyte anterior during stages 3-6 [41]. With this in mind, the high levels of *Wolbachia* in cystocytes during differentiation into oocyte and nurse cells in region 2a of the germarium may possibly lead to impairment at the structural and/or molecular level, the cyst may undergo apoptosis as a consequence (Figure 7B).

On the other hand, the increase in the number of germaria containing apoptotic cysts may result from the action of the bacteria on the SSCs, which gives rise to follicle cells in region 2b of the germarium (Figure 7A, C). Drummond-Barbosa and Spradling [8] have suggested that apoptosis in region 2a/2b of the germarium serves to maintain the proper ratio of germline cells to somatic follicle cells. In poorly fed flies, follicle cells slow down their proliferation, the germline cells to somatic follicle cell ratio becomes skewed, resulting in cyst apoptosis in region 2a/2b which corrects this ratio [8]. It has been established that stem cells are maintained in specialized microenvironment called the niche [42]. The abundance of *Wolbachia* in the SSCN [26] is of interest in this context. Thus reasoning, it may be assumed that the presence of *Wolbachia* in the SSCN decreases the SSC proliferation rate, the ratio of germline cells to follicle cells becomes imbalanced and, as a consequence, cysts undergo apoptotic death. Judging from our current data, the ultrastructural appearance of follicle cells in region 2b of the germarium from ovaries of wMelPop-infected *D. melanogaster^w1118^* was normal, thereby indicating that *Wolbachia* presumably did not negatively affect follicle cells. It should be noted that the fecundity of the wMelPop infected *D. melanogaster ^w1118^* was not decreased as compared with their uninfected counterparts [43,44]. This was evidence of insect plasticity, rendering them capable to adapt to diverse factors.

Taken together, our findings clearly demonstrated that the *Wolbachia* strain wMelPop has an effect on the egg chamber formation in the *D. melanogaster* germarium. However, the underlying mechanism is still unclear. We intend to perform a comparative morphometric analysis of apoptotic structures and bacteria in cystocytes of wMel- and wMelPop-infected flies. The results would be helpful in deciding whether the increase in apoptosis frequency is due to high bacterial density or to particular pathogenic effect of the *Wolbachia* strain wMelPop on female germline cells.

## Conclusions

The results of this study showed that the presence of the *Wolbachia* strain wMelPop in *D. melanogaster* ovaries led to an increase in the frequency of apoptosis in the germarium checkpoint. Two possible pathways along which *Wolbachia* affect egg chamber formation in region 2a/2b of the germarium have been suggested. Future research should be conducted to clarify the mechanism underlying this phenomenon.

## Methods

### *Drosophila* stocks and maintenance

The *Drosophila melanogaster* Canton S infected with the *Wolbachia* strain wMel (IC&G, Russia) and *D. melanogaster^w1118^* infected with wMelPop (a kind gift from prof. S. O’Neill, The University of Queensland, Australia) were used in these experiments. Flies were maintained at 25 °C either on a standard yeast-agar medium or on daily replaced rich food (standard medium covered with wet yeast paste). To obtain uninfected *D. melanogaster^w1118T^*, flies were raised on food supplemented with tetracycline at 0.03% for two generations, then on standard food for more than three generations [43]. Confirmation of the infection status of each stock was provided by PCR. For this purpose, total DNA extracted from fly ovaries and *wsp* 81F/*wsp* 691R primers for amplifying a *Wolbachia* surface protein gene fragment were used [45].

### Acridine orande staining

Acridine orange (AO), a vital stain highly specific to apoptotic nuclei, was used [46]. Ovaries were dissected from 5-day old flies in EBR buffer (130 mM NaCl, 4.7 mM KCl, 1.9 mM CaCl_2_, 10 мM Hepes pH 6.9), stained with AO (Merck), 5 μg/ml, in 0.1 M sodium phosphate buffer, pH 7.2, for 3 min at room temperature [12,47]. Samples were placed onto glass slides and covered with halocarbon oil (KMZ Chemicals Ltd.). They were viewed under an Axioscop 2 plus fluorescence microscope (Zeiss) using an appropriate filter (Zeiss filter set 02). Time elapsed from dissection to the end of viewing was restricted, 20 min. Staining of nuclei varied from bright yellow to brilliant orange, depending on the stage of degeneration [46]. The percentage of AO-staining germaria was expressed as the ratio of the number of AO-stained germaria containing apoptotic cells to the total number of analysed germaria. Three experiments were performed for each of the 4 *D. melanogaster* groups (w1118, w1118T stocks, standard food; w1118, w1118T, rich food). In each replicate, ovaries were dissected from 6 flies, 7-12 germaria per fly were analysed. In all, about 1350 AO-stained germaria were analysed. Bartlett’s test was used to check homogeneity of variances. Two-way ANOVA was used to determine the significance of the difference between the frequency of apoptosis of the uninfected and *Wolbachia*-infected flies maintained on different food.

### TUNEL assay

TUNEL was the independent assay of detection of apoptotic cells. TUNEL is advantageous because preferentially labeling apoptotic cells relatively late in the apoptotic process [48]. Ovaries were dissected from 5-day old flies in phosphate-buffered saline (PBS), fixed in PBS containing 4% formaldehyde plus 0.1% Triton X-100 for 25 min. Then, they were separated into individual ovarioles, rinsed briefly in PBS twice and washed in PBS three times for 5 min each. Ovarioles were made permeable with 20 μg/ml proteinase K in PBS for 20 min at room temperature, this was followed by 3 washes in PBS for 5 min each. The TUNEL reaction and all the subsequent steps were performed using the FragEL DNA Fragmentation Detection Kit (Calbiochem) according to the manufacturer’s protocol. Samples were viewed with an Axioscop 2 plus fluorescent microscope (Zeiss), images were captured with a high resolution microscopy camera AxioCam HRc and AxioVision software. Germaria from ovaries of 10 flies were counted in each of the 4 groups. The total number of germaria analysed was about 850. The data were compared using a Chi-square test (χ^2^).

### Electron microscopy

Fixation of the *D. melanogaster* ovaries was carried out using the method described previously [49,35]. Briefly, 5 day-old females were dissected in 0.1 M phosphate buffer, pH 7.4, fixed in 2.5% glutaraldehyde (Sigma) in 0.1 M sodium cacodylate buffer, pH 7.4, for 2.5 h. This was followed by washings in the same buffer and postfixation in 1% OsO_4_ and 0.8% potassium ferrocyanide for 1 h. After washings, samples were placed in 1% aqueous solution of uranyl acetate (Serva) for 12 h at 4 °C. Then they were dehydrated in ethanol series and acetone, finally samples were embedded in Agar 100 Resin (Agar Scientific Ltd.). Ultra-thin sections were stained with uranyl acetate and Reynolds lead citrate. They were examined with a transmission electron microscope (JEM 100 SX, JEOL). The number of flies analysed in each of the 4 groups was 8-12.

## Authors' contributions

MZ performed the experiments. EK and MZ both designed the study, drafted and wrote the manuscript. Both authors have read and approved the final text.

## Competing interests

The authors declare that they have no competing interests.

## Supplementary Material

Additional file 1 **TUNEL in the germaria from ovaries of *D. melanogaster.* Three groups of germaria are distinguished.** A, B, the TUNEL-negative germaria from the ovaries of *D. melanogaster^w1118T^* and Canton ST, respectively. C, D, the TUNEL-positive germaria with 1-2 distinct puncta in region 2a/2b of the germarium from the same fly stocks, as in A, B. E, F, the TUNEL-positive germaria with clusters of bright spots. Region 2a/2b of the germarium is indicated by red brackets. Scale bars: 20 μm. Click here for file

Additional file 2 **Cystocytes in region 2a/2b of the germarium from the wMel-infected *D. melanogaster* Canton S.** Bacteria with moderate density matrix are indicated by black arrows; white arrow points to a bacterium with light matrix; ring canals between cystocytes are marked by arrowheads. Scale bar: 2 μm. Click here for file

Additional file 3 **Morphology of apoptotic cystocytes in region 2a/2b of the germaria from the uninfected *D. melanogaster^w1118T^*.** A, cyst cells containing swollen mitochondria (arrows). B, a normal mitochondrium (arrowhead) and swollen mitochondria in the cytoplasm of a cyst cell. C, pyknotic nuclei in cyst cells. D, an apoptotic body (ab) containing remnants of a fragmented cell. Scale bars: 1 μm. Click here for file

Additional file 4 **The *Wolbachia* strain wMel in cyst cells undergoing apoptosis in region 2a/2b of the germaria.** A, apoptotic cystocytes, low magnification view. B, bacteria framed in panel A depicted at higher magnification. Bacteria showing normal morphology (arrows), with light matrix (white arrowheads), with light matrix and disrupted envelope (black arrowheads) in the cytoplasm of dying cell. Scale bars: 2 μm. Click here for file

Additional file 5 **Follicle cells in region 2b of the germaria from wMelPop-infected *D. melanogaster^w1118^*.** A, follicle cells containing small amounts of bacteria (arrows). B, follicle cells and apoptotic cyst cells (ac). Scale bars: 2 μm. Click here for file

Additional file 6 **Ultrastructure of germarium cells at periphery of region 1 in wMel-infected *D. melanogaster* Canton S.** A, B, fragments of cells whose cytoplasm contains numerous autophagosomes, bacteria and multilayered membranes (low magnification view). C, high-magnification micrograph of the fragment shown in panel A (framed) demonstrating a bacterium enclosed by autophagosome. D-F, autophagosomes containing numerous membranes and inclusions varying in electron density. Scale bars correspond to 1 μm (A, B) and 0.5 μm (C-F), respectively. Click here for file
