# Supplementary figures and images for: The virulent Wolbachia strain wMelPop increases the frequency of apoptosis in the female germline cells of Drosophila melanogaster
Source: BMC Microbiol. 2012 Jan 18;12(Suppl 1):S15. doi: 10.1186/1471-2180-12-S1-S15 (PMC3287512; doi:10.1186/1471-2180-12-S1-S15)

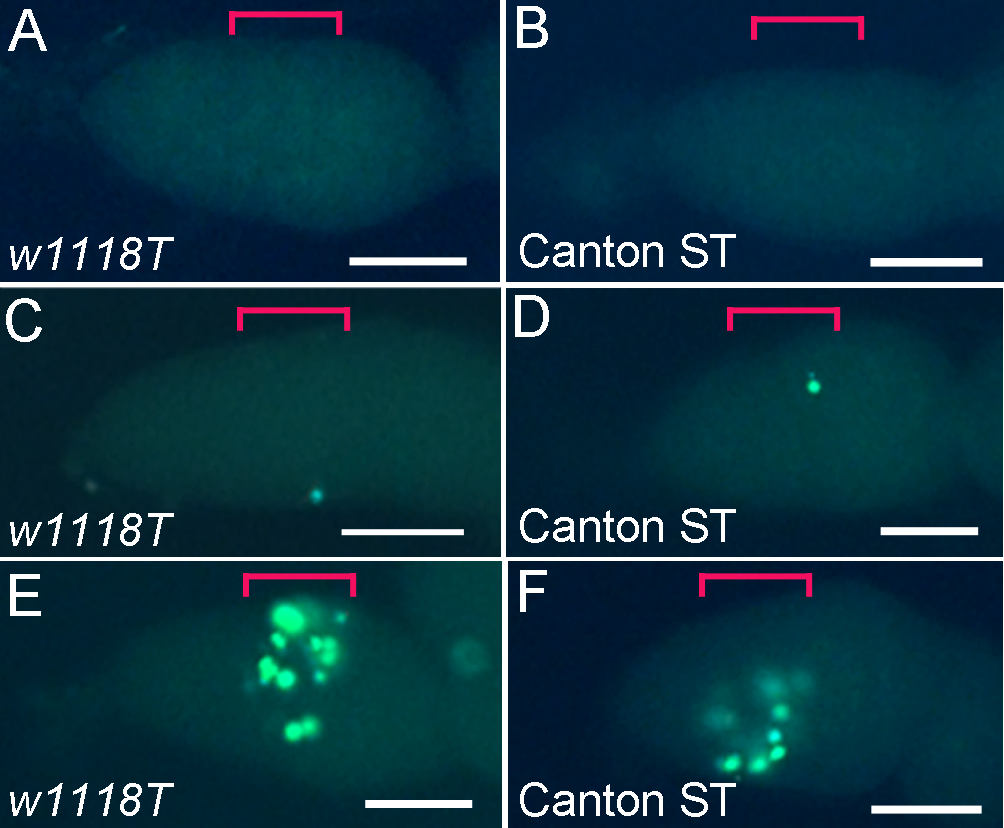

Supplement: Additional file 1 — TUNEL in the germaria from ovaries of D. melanogaster. Three groups of germaria are distinguished. A, B, the TUNEL-negative germaria from the ovaries of D. melanogasterw1118T and Canton ST, respectively. C, D, the TUNEL-positive germaria with 1-2 distinct puncta in region 2a/2b of the germarium from the same fly stocks, as in A, B. E, F, the TUNEL-positive germaria with clusters of bright spots. Region 2a/2b of the germarium is indicated by red brackets. Scale bars: 20 μm. [file 1471-2180-12-S1-S15-S1.tif]

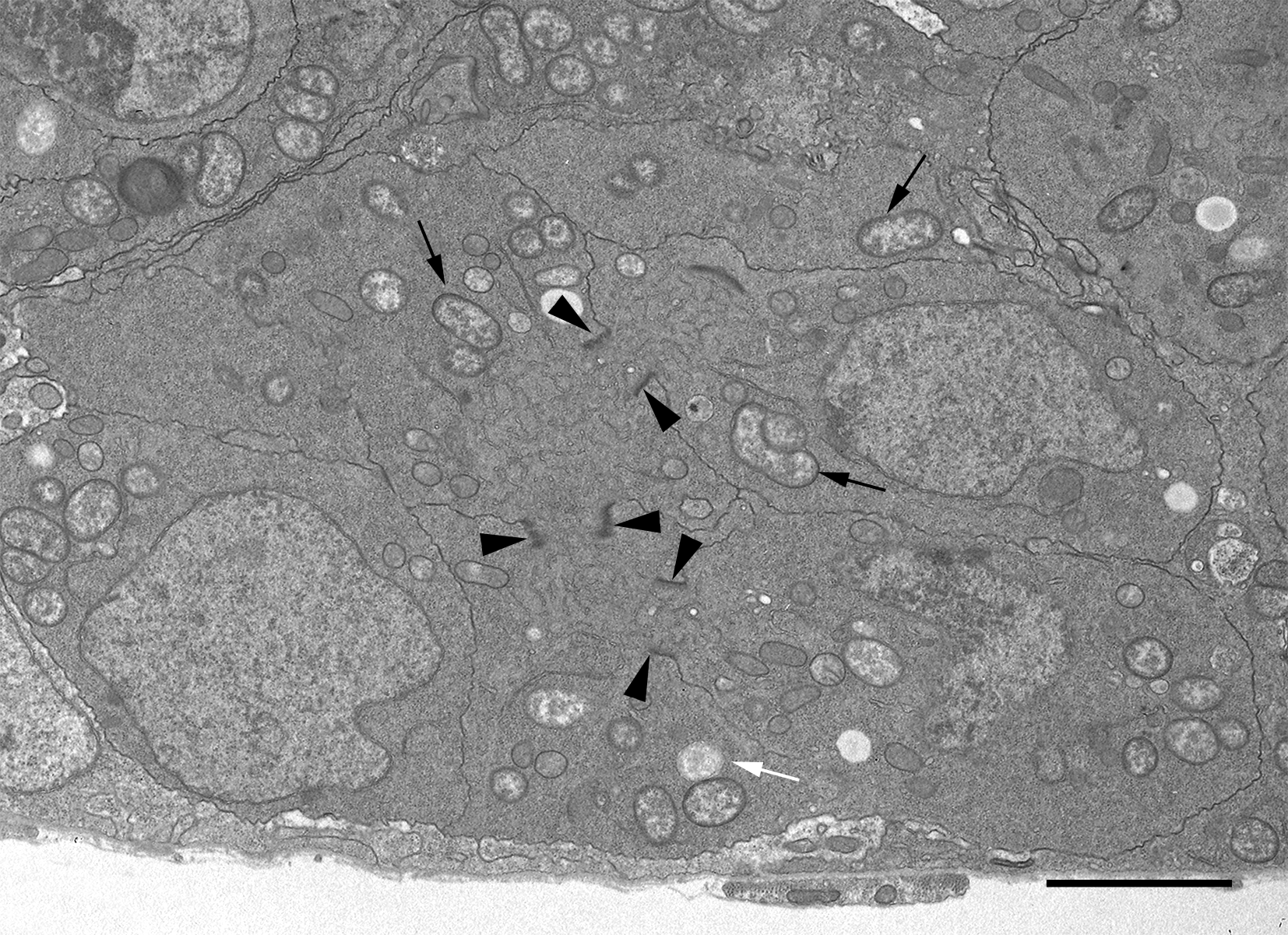

Supplement: Additional file 2 — Cystocytes in region 2a/2b of the germarium from the wMel-infected D. melanogaster Canton S. Bacteria with moderate density matrix are indicated by black arrows; white arrow points to a bacterium with light matrix; ring canals between cystocytes are marked by arrowheads. Scale bar: 2 μm. [file 1471-2180-12-S1-S15-S2.tif]

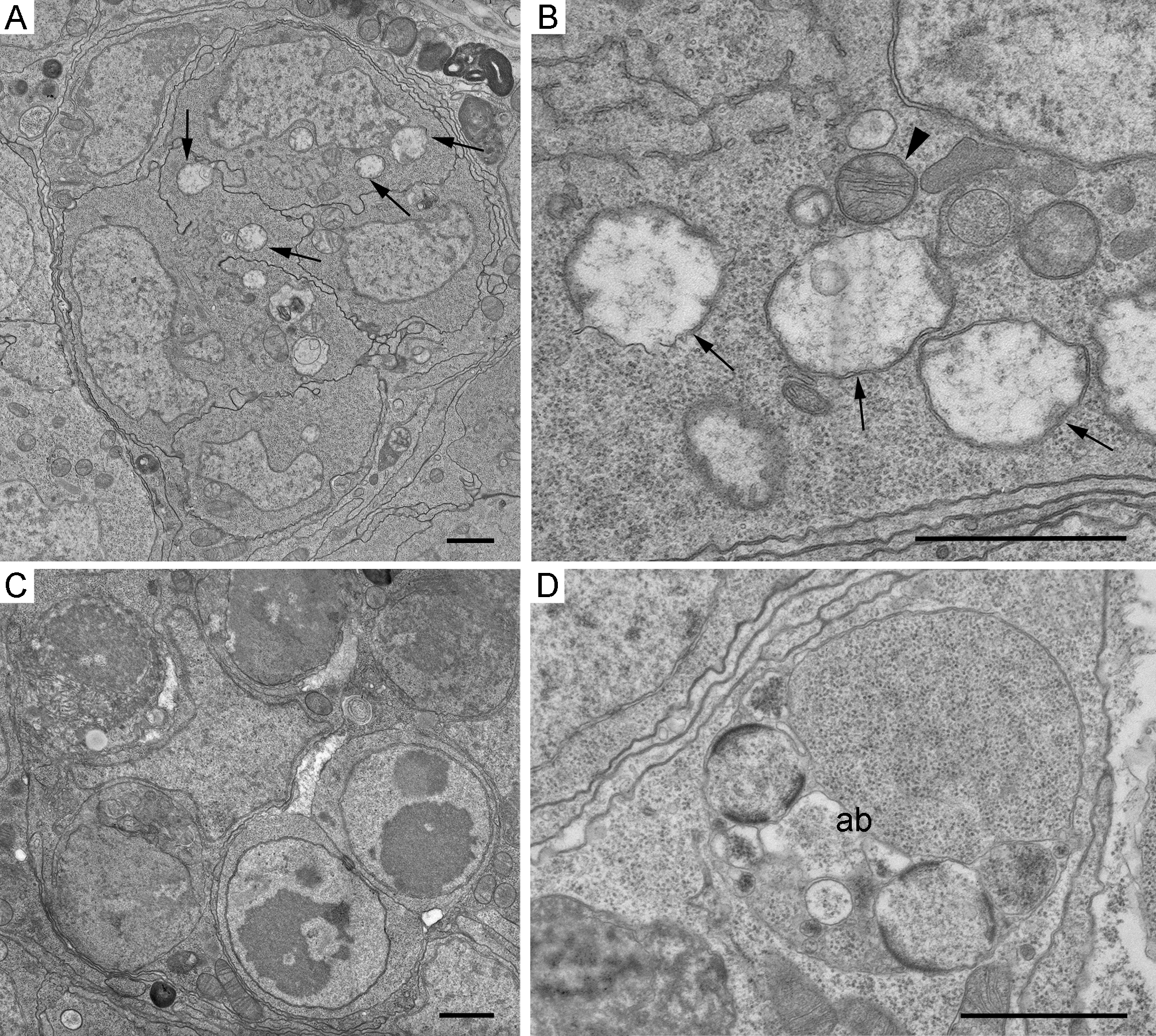

Supplement: Additional file 3 — Morphology of apoptotic cystocytes in region 2a/2b of the germaria from the uninfected D. melanogasterw1118T. A, cyst cells containing swollen mitochondria (arrows). B, a normal mitochondrium (arrowhead) and swollen mitochondria in the cytoplasm of a cyst cell. C, pyknotic nuclei in cyst cells. D, an apoptotic body (ab) containing remnants of a fragmented cell. Scale bars: 1 μm. [file 1471-2180-12-S1-S15-S3.tif]

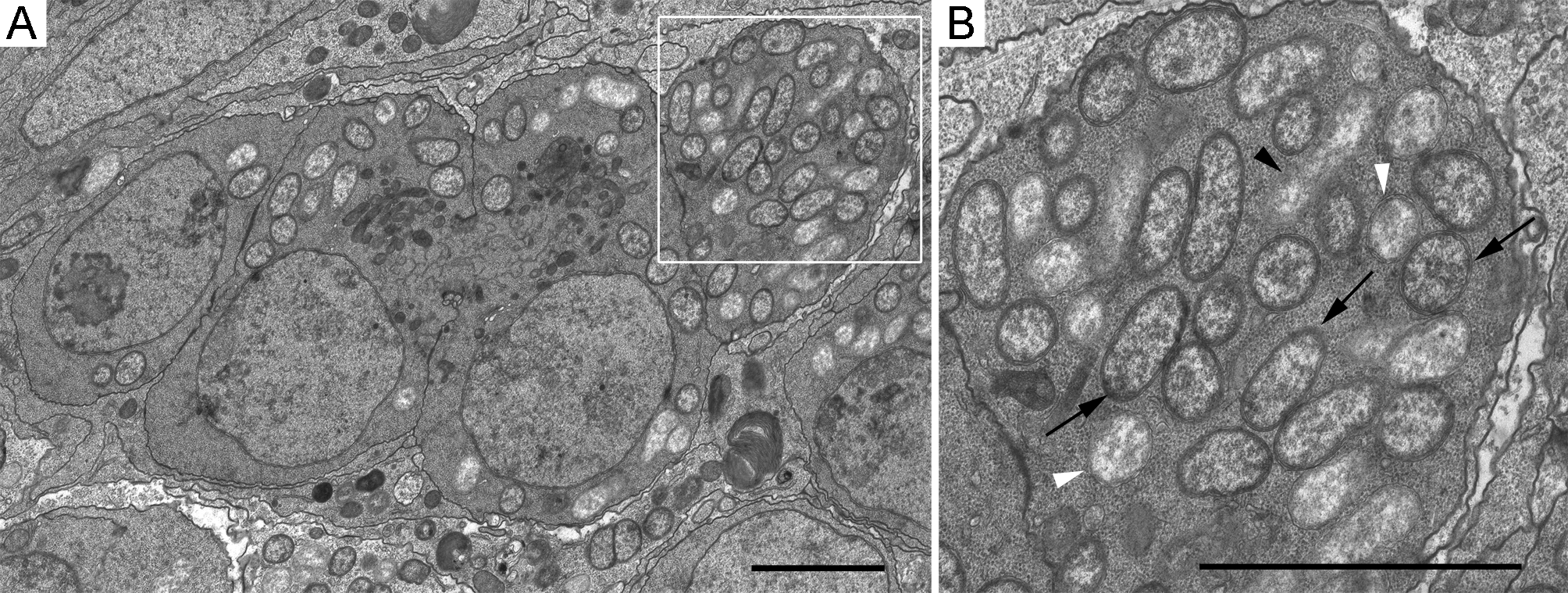

Supplement: Additional file 4 — The Wolbachia strain wMel in cyst cells undergoing apoptosis in region 2a/2b of the germaria. A, apoptotic cystocytes, low magnification view. B, bacteria framed in panel A depicted at higher magnification. Bacteria showing normal morphology (arrows), with light matrix (white arrowheads), with light matrix and disrupted envelope (black arrowheads) in the cytoplasm of dying cell. Scale bars: 2 μm. [file 1471-2180-12-S1-S15-S4.tif]

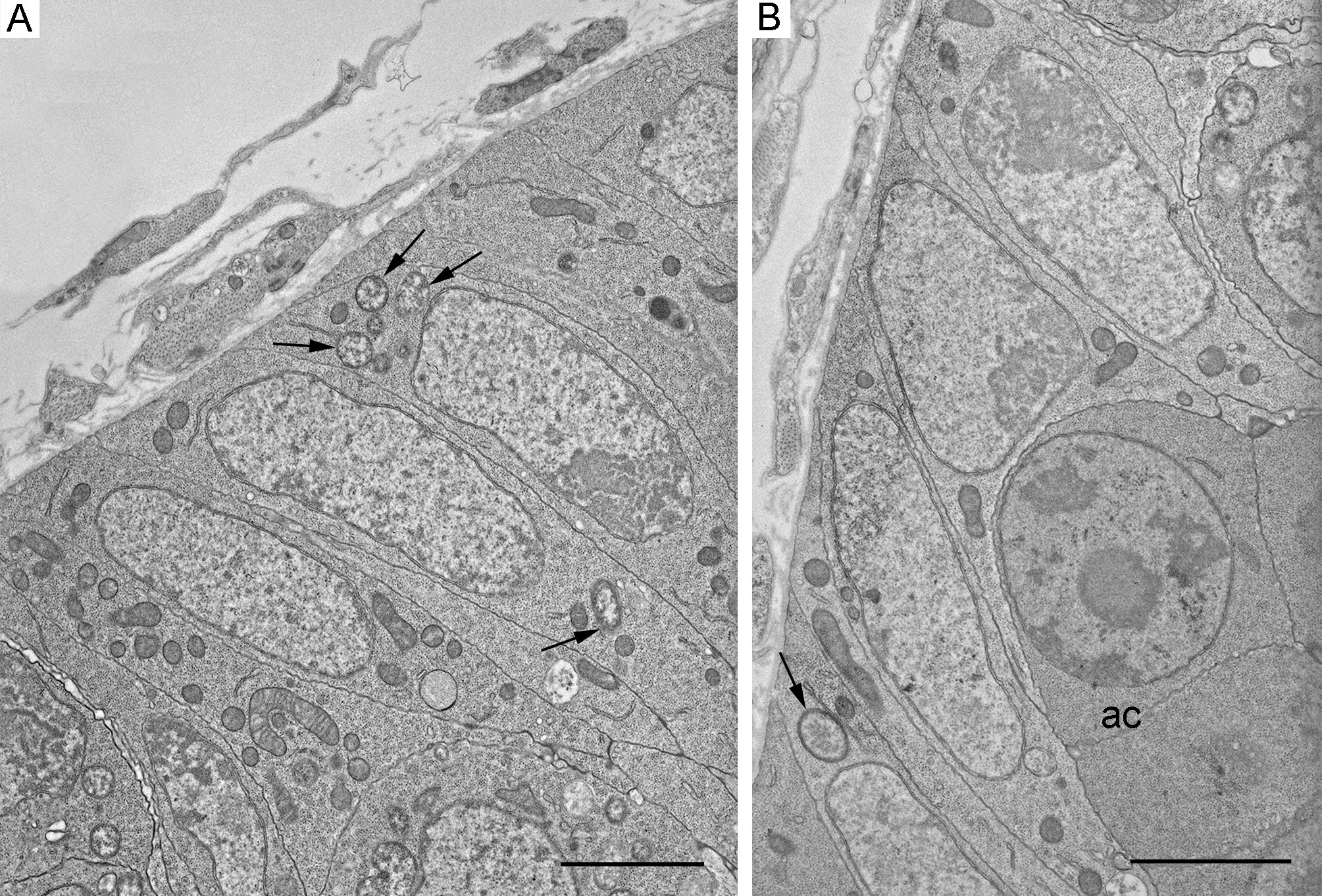

Supplement: Additional file 5 — Follicle cells in region 2b of the germaria from wMelPop-infected D. melanogasterw1118. A, follicle cells containing small amounts of bacteria (arrows). B, follicle cells and apoptotic cyst cells (ac). Scale bars: 2 μm. [file 1471-2180-12-S1-S15-S5.tif]

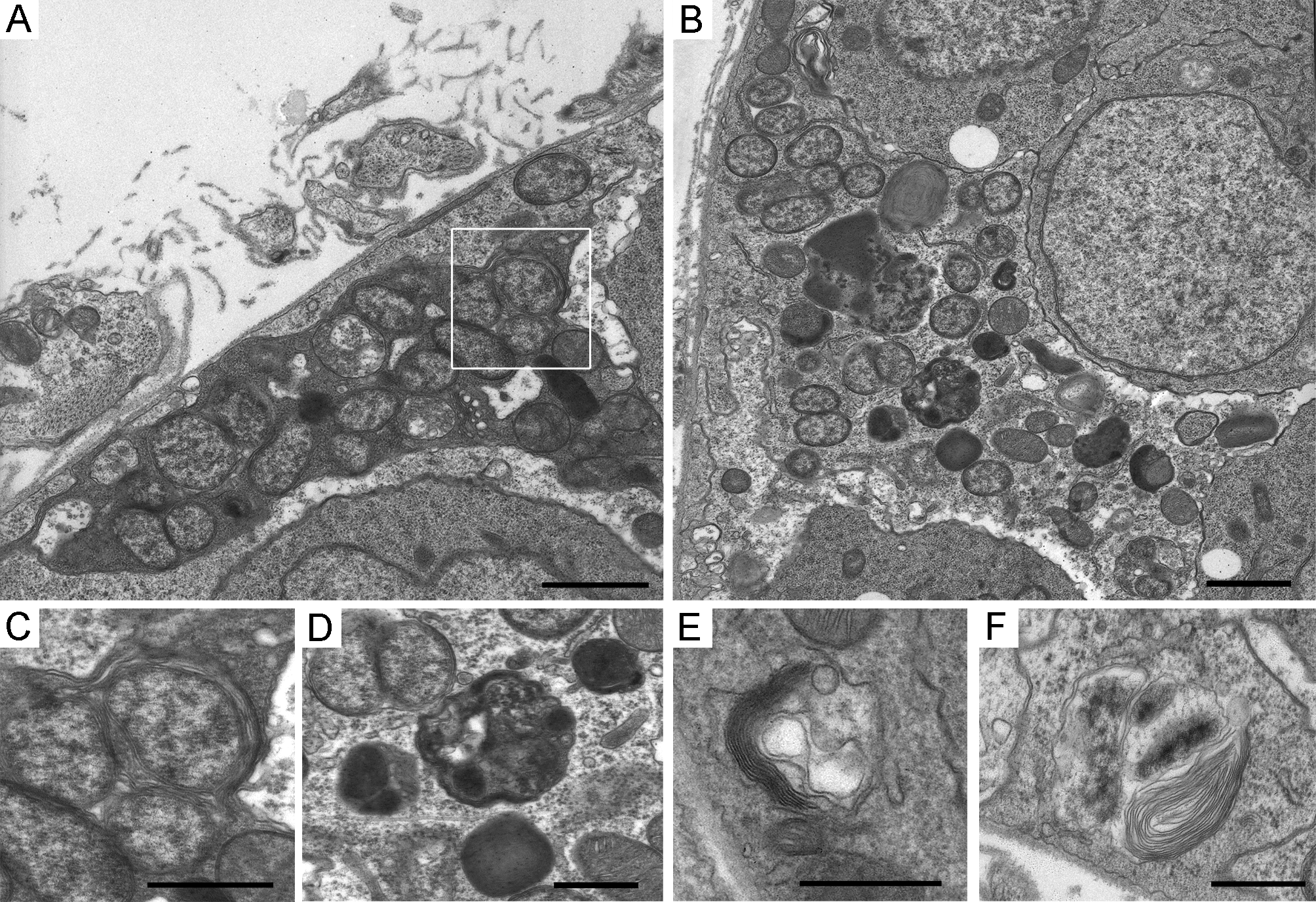

Supplement: Additional file 6 — Ultrastructure of germarium cells at periphery of region 1 in wMel-infected D. melanogaster Canton S. A, B, fragments of cells whose cytoplasm contains numerous autophagosomes, bacteria and multilayered membranes (low magnification view). C, high-magnification micrograph of the fragment shown in panel A (framed) demonstrating a bacterium enclosed by autophagosome. D-F, autophagosomes containing numerous membranes and inclusions varying in electron density. Scale bars correspond to 1 μm (A, B) and 0.5 μm (C-F), respectively. [file 1471-2180-12-S1-S15-S6.tif]
